# Prognostic value of cardiovascular magnetic resonance in acute myocarditis: a systematic literature review and meta-analysis of late gadolinium enhancement and narrative literature review of parametric mapping

**DOI:** 10.1186/s12880-026-02623-6

**Published:** 2026-07-31

**Authors:** Karolina Gaizauskiene, Viktor Skorniakov, Dovile Silinskiene, Nomeda Valeviciene, Sigita Glaveckaite

**Affiliations:** 1 Department of Radiology, Nuclear Medicine and Medical Physics, Institute of Biomedical Sciences, Vilnius University Faculty of Medicine, Santariskiu st. 2, Vilnius, 08661 Lithuania; 2 Faculty of Mathematics and Informatics, Institute of Applied Mathematics, Vilnius University, Naugarduko str. 24, Vilnius, LT-03225 Lithuania; 3 Vilnius University Hospital Santaros Klinikos, Santariskiu st. 2, Vilnius, 08661 Lithuania; 4 Clinic of Cardiovascular Diseases, Institute of Clinical Medicine, Faculty of Medicine, Vilnius University, Santariskiu st. 2, Vilnius, 08661 Lithuania

**Keywords:** Cardiovascular magnetic resonance, Prognostic value, Acute myocarditis, Late gadolinium enhancement, Parametric mapping, T1 mapping, T2 mapping, Extracellular volume

## Abstract

**Background and aims:**

Limited data exist on the prognostic value of parametric mapping and late gadolinium enhancement (LGE) in patients with acute myocarditis (AM). This meta-analysis aimed to investigate the prognostic value of LGE as assessed by using cardiovascular magnetic resonance (CMR) in patients with AM, including more recent evidence. Additionally, the prognostic value of CMR parametric mapping was narratively reviewed.

**Materials and methods:**

The systematic search was conducted in the PubMed and Scopus databases from inception to January 17, 2026 (PubMed) and January 20th 2026 (Scopus). 1296 records were found through the search. Finally, sixteen studies were included in this systematic review. Thirteen studies were included in the meta-analysis on association between LGE and clinical outcomes in AM. The composite endpoint was defined as MACE and/or one of the following endpoints: all-cause death, heart failure decompensation requiring hospitalization, heart transplantation, and recurrent AM. MACE was defined as a composite of cardiovascular death (including sudden death), sustained ventricular tachycardia, ventricular fibrillation, complete atrioventricular heart block requiring a pacemaker, and cardiogenic shock. Five studies were reviewed for the prognostic value of parametric mapping parameters. After initial calculations, it appeared that studies using broader composite endpoints than only MACE introduced substantial heterogeneity. Therefore, we have additionally conducted analysis incorporating only eight studies with MACE as the endpoint.

**Results:**

The presence of LGE was associated with higher odds of the composite endpoint. The unadjusted pooled OR demonstrated a significant relationship between the presence of LGE and higher chances of the composite endpoint (OR_LGE(+) vs. LGE(−)_[95% CI] = 3.02 [1.40, 6.51], *p* < 0.001) with high heterogeneity (I^2^ = 81.98%, *p* < 0.0001) On the qualitative level, results obtained in the MACE studies subgroup remained the same (OR_LGE(+) vs. LGE(−)_[95% CI] = 2.88 [2.01, 4.13], *p* < 0.0001). No clear evidence of publication bias was detected; however, power to detect small-study effects is limited and results should be interpreted cautiously. The prognostic value of parametric mapping parameters did not undergo meta-analysis and was reviewed narratively due to the shortage of the respective data. The data on the prognostic value of native T1, T2, and extracellular volume (ECV) are controversial and limited.

**Conclusions:**

Based on the findings, the presence of LGE appears to be associated with an adverse prognosis in patients with AM. However, these findings should be interpreted with caution because of substantial heterogeneity among studies and the unadjusted effect estimations. Evidence regarding the prognostic value of CMR-derived parametric mapping remains limited. Large, prospective, well-designed studies are needed to determine the incremental prognostic value of parametric mapping beyond LGE for risk stratification and outcome prediction in patients with AM.

**Supplementary Information:**

The online version contains supplementary material available at https://doi.org/10.1186/s12880-026-02623-6.

## Introduction

Myocarditis is an inflammatory disease of the myocardium caused by various triggers. It is predominantly caused by viruses but can also be triggered by drugs, toxic substances, or autoimmune conditions [1, 2].

Myocarditis is a challenging diagnosis to make, with a heterogeneous clinical presentation, natural history, and prognosis. Symptoms range from no or mild symptoms to chest pain, dyspnoea, fatigue, palpitations, syncope, cardiogenic shock, and sudden cardiac death (SCD) [1].

In clinical practice, cardiovascular magnetic resonance (CMR) has emerged as a diagnostic tool to confirm clinically suspected acute myocarditis (AM) [3]. CMR is the gold standard imaging technique for assessing cardiac function and accurately measuring ventricular volumes and mass [4]. Contemporary multiparametric CMR has a high diagnostic yield in patients with suspected AM [1, 5, 6].

Previous studies reported that late gadolinium enhancement (LGE) is a strong predictor of unfavourable outcomes and could be used for risk stratification in hypertrophic cardiomyopathy (HCM), dilated cardiomyopathy (DCM), or non-ischemic cardiomyopathy [7, 8]. Only a few studies have addressed the prognostic value of CMR parameters in myocarditis [1].

This systematic literature review and meta-analysis aimed to investigate the impact of the presence of LGE on a composite endpoint consisting of major adverse cardiovascular events (MACE) and/or one of the following endpoints: all-cause death, heart failure decompensation requiring hospitalization, heart transplantation, and recurrent AM. MACE was defined as a composite of cardiovascular death (including sudden death), sustained ventricular tachycardia and/or ventricular fibrillation, complete atrioventricular heart block requiring a pacemaker implantation, and cardiogenic shock. Present meta-analysis included newer studies not being analysed in previous publications on the topic. Another goal was to narratively review the prognostic value of CMR parametric mapping parameters, which hasn’t been analysed in the available literature.

## Materials and methods

### Data sources and search strategy

This systematic review and meta-analysis were performed using the Preferred Reporting Items for Systematic Reviews and Meta-Analyses (PRISMA) statement [9]. The systematic search was conducted in the PubMed and Scopus databases by two independent investigators (K.G. and D.S.) from inception to January 17, 2026 (PubMed) and January 20th 2026 (Scopus) using the following free-text keywords related to myocarditis, prognosis, and cardiac magnetic resonance imaging in combination with Boolean operators (AND, OR). No MeSH terms were applied. PubMed was searched in all fields. The exact PubMed query: ((“Myocarditis”) AND (“outcome*” OR “prognostic value” OR “prognosis”) AND (“Cardiovascular magnetic resonance” OR “CMR” OR “cardiac magnetic resonance” OR “late gadolinium enhancement” OR “delayed gadolinium enhancement” OR “LGE” OR “mapping” OR “T1” OR “T2” OR “ECV” OR “extracellular volume”)). In Scopus database keywords: “Myocarditis” “outcome*” OR “prognostic value” OR “prognosis” and “Cardiovascular magnetic resonance” OR “CMR” OR “cardiac magnetic resonance” OR “late gadolinium enhancement” OR “delayed gadolinium enhancement” OR “LGE” OR “mapping” OR “T1” OR “T2” OR “ECV” OR “extracellular volume” were searched specifically in the Abstract field only combining by AND. No language, publication date, document type, or other filters or limits were applied in either database. Any disagreements were resolved through consensus discussion between the two reviewers.

### Study selection

Titles and abstracts were screened to determine study eligibility using the following pre-determined inclusion criteria:

1. adult patients’ studies with clinically suspected AM;
2. CMR at 1.5 T or 3 T field strength;
3. qualitative or quantitative reporting of at least one CMR parameter of interest, namely LGE, T1 mapping time, T2 mapping time, or extracellular volume (ECV);
4. outcomes comparison of LGE with and non-LGE;
5. studies written in English.

We excluded reviews, case reports, editorial comments, meta-analyses, articles in non-English languages, and studies without insights into prognosis.

This meta-analysis included all studies describing prognostic value of LGE detected by CMR in adult patients with suspected AM. The total number of events, including MACE, and/or all-cause mortality, heart failure decompensation requiring hospitalization, heart transplantation, and recurrent acute myocarditis, stood for this meta-analysis’s combined events. We have also conducted additional analysis incorporating only studies reporting major adverse cardiac events (MACE) as an outcome. MACE was chosen to capture clinically definitive and robust outcomes. Studies using composite or only all-cause death endpoints were excluded to mitigate observed heterogeneity and to inspect whether, in this homogeneous study population, qualitative conclusions remained the same.

The prognostic value of parametric mapping parameters did not undergo meta-analysis and was reviewed narratively due to the shortage of the respective data.

### Data collection

The data extracted from the eligible publications included the first author’s name, type of study, sample size, mean age, the proportion of males, magnet field strength, the reference standard for the diagnosis of myocarditis, days between symptom onset and CMR performed, median follow-up duration, the number of combined events, and CMR parameters analysed in the study. The extracted CMR parameters were LGE, T1 mapping time, T2 mapping time, and ECV.

### Quality of included studies

The Newcastle–Ottawa Quality Assessment Scale was used to assess the quality of the included publications [10]. Quality assessment was performed only for studies included in the quantitative meta-analysis. In addition to the overall scores, we evaluated key risk-of-bias domains, including patient selection, comparability of groups, outcome assessment, completeness of follow-up, and selective reporting. Each domain was rated as low, moderate, or high risk of bias. All 13 studies included in the meta-analysis scored 6 or higher, indicating high quality (Supplementary files, Table S1).

### Statistical analysis

Statistical analysis was done using the R language [11] and the R-Studio [12] environment for statistical computing (version 4.3.3). Package metafor (version 4.8-0) [13] was used to perform meta-analysis-related tasks. Random effects (RE) model based on the Restricted-Maximum-Likelihood Estimator was applied to compute pooled estimators [14, 15]. Forest plots were used to summarize and present the pooled analysis results. Pooled unadjusted OR estimates were derived from 2 × 2 tables representing event counts. Adjusted ORs, though present in many studies, were not used for pooling because of (1) varying sets of covariates and (2) different methodological approaches employed by different studies for estimation of ORs. Effects’ heterogeneity was measured by the magnitude of I^2^ value, corresponding test, and funnel plots. Alternative Fixed Effects (FE) model was not considered because of the reason [13, 16] that analysis based on FE model is limited to the studies included and does not generalize to other similar studies. An investigation of publication bias was made using Linear Egger’s test [17]. To identify potentially influential studies, leave-one-out analysis was used, accompanied by cumulative meta-analysis, which provided insight into the temporal effect’s dynamics. The significance level is assumed to be equal to 0.05; all reported confidence intervals (CIs) are 95% CIs.

## Results

### Search results

**Fig. 1:**
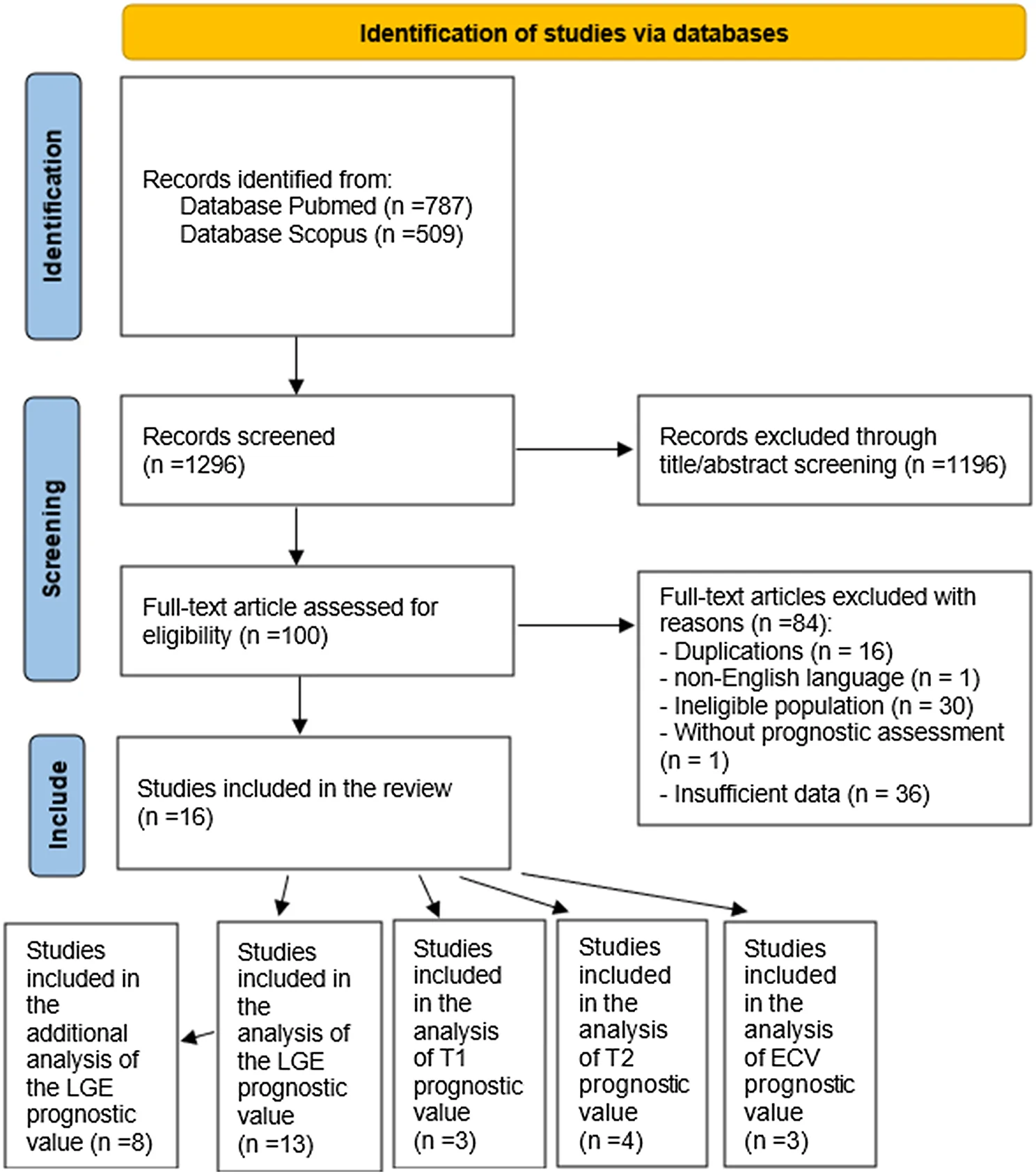
Flowchart of the study

Through our search, 1296 records were identified, and all titles/abstracts were reviewed for inclusion. No filters/limits were applied except of final date of 20th of January 2026. Based on the inclusion/exclusion criteria, 1196 were excluded, and the remaining 100 were screened for eligibility. Finally, 16 studies meeting all inclusion and exclusion criteria were included in this systematic review (Table 1). We excluded one study from meta-analysis on ICI myocarditis as it represents a distinct clinical condition with different pathophysiological mechanisms, histological characteristics, and prognosis [18]. 13 studies were included in the meta-analysis to calculate pooled ORs of LGE presence [19–31]. After initial calculations, it appeared that studies focusing not only on MACE but also including all-cause death introduced substantial heterogeneity. Therefore, we have conducted additional analysis incorporating only 8 MACE studies [20–25, 30, 31]. Five studies were analysed for the significance of parametric mapping parameters [21, 23, 30, 32, 33]. The flowchart of the study is shown in Fig. 1.

### Characteristics of studies analysed

All the studies were published between 2012 and 2026. Fourteen were retrospective, while two were prospective [23, 26]. Most were single-centre studies, while six were multicentre [18, 21, 24, 27, 28, 31], and one included two centres [32]. Two studies used endomyocardial biopsy (EMB) to confirm the diagnosis of AM. In one study, the diagnosis was confirmed by diagnostic CMR criteria or endomyocardial biopsy, while the diagnosis in the remaining ten studies was based only on clinical or CMR criteria. Three studies included patients with Immune Checkpoint Inhibitor–Associated Myocarditis (ICI-myocarditis). The patient’s mean follow-up duration was 3.7 years.

Fourteen studies used a 1.5 T magnet; five used 1.5 and 3 T; and two used 3 T (Table 1). The analysed studies reported CMR findings on LGE (13 studies [19–31]), T1 mapping (3 studies [30, 32, 33]), T2 mapping (4 studies [23, 30, 32, 33]), and ECV (3 studies [21, 30, 32]). Table 1 presents the characteristics of the included studies.

**Table 1:** Characteristics of the included studies and patients’ clinical characteristics

First author, study year	No of patients	CMR strength, T	Timing of CMR from symptom onset (days)	Median follow-up duration, years	Population	Age, years	Male gender, *n* (%)
Cadour et al. (2022) [32]	33	1.5 and 3	In two weeks	92 days	ICI - myocarditis	68 ± 14	23 (69.7)
Thavendiranathan et al. (2021) [33]	86	1.5 and 3	NA	158 days	ICI - myocarditis	66.3 ± 13.1	58 (67.4)
Zhang et al. (2020) [18]	103	1.5 and 3	In 4 or later	148.5 (62–304) days	ICI - myocarditis	65.6 ± 15.3	73 (70.9)
Greulich et al. (2020) [19]	183	1.5	5	10.1	Biopsy-Proven Viral myocarditis	53 (40–67)	126 (69)
Gräni et al. (2019)0.1 [20]	670	1.5 and 3	NA	4.7	Clinically suspected AM	48 ± 16	392 (59)
Gräni et al. (2019)0.2 [21]	179	3	0–5	4.1	Suspected AM	49 ± 15	100 (56)
Lee et al. (2017) [22]	37	1.5 and 3	11.6 ± 8.5	41 mo	Suspected AM	41.5 ± 17.5	22 (59.5)
Spieker et al. (2017) [23]	46	1.5	NA	Mean 11 ± 7 mo	Suspected AM	41 ± 16	33 (72)
Aquaro et al. (2017) [24]	374	1.5	NA	4.3	AM with preserved LVEF	35 ± 15	299 (73)
Chopra et al. (2016) [25]	88	1.5	NA	1.3	CMR-based diagnosis of AM	39.2 ± 16.4	70 (80)
Schumm et al. (2014) [26]	405	1.5	14	4.4	Clinically suspected AM	47.9 (36.9–60.8)	228 (56.3)
Grün et al. (2012) [27]	203	1.5	5	4.7	Biopsy-proven viral myocarditis	52 (40–54)	140 (69)
Vos et al. (2022) [28]	162	1.5	6 (3–9)	5.5 (2.2–8.3)	CMR-based diagnosis of AM	41 ± 17	121(75)
Ghionzoli et al. (2022) [29]	61	1.5	Within 3	4.8 ± 0.6	AM with preserved LVEF	39 ± 12	49(80)
Wang et al. (2025) [30]	235	3	Median 13	1637 days	CMR-based or biopsy-proven AM	32 ± 13	150 (63.8)
Aquaro et al. (2026) [31]	210	1.5	NA	57 months	Clinically suspected AM	42(22–55)	164(78)

Mo – months, NA – data not available

### Patients’ clinical characteristics

The systematic review and meta-analysis include a group of 3075 patients with suspected or biopsy-proven AM. The mean age of the patients was 47.53 years, with 66.6% being male. The patients’ clinical characteristics are shown in Table 1.

### Association between LGE and clinical outcomes

Data concerning the numbers of patients with and without adverse events, stratified by the presence or absence of LGE, were extracted from the studies (Table 2a, 2b).

**Table 2a:** Summary of studies with available prognostic data in relation to the presence of LGE on CMR and parametric mapping parameters

First author, study year	No of patients	Endpoints	LGE (+), n	Total n of events	Total number of events, LGE (+)	Total number of events, LGE (-)	T1 elevated, n (%)	T2 elevated, n (%)	ECV elevated, n (%)	Total n of events (%)
Cadour et al. (2022) [32]	33	MACE*: cardiovascular death, documented sustained (.30 seconds) ventricular tachycardia, ventricular fibrillation, complete atrioventricular (AV) heart block, and cardiogenic shock	NA	NA	NA	NA	9 (36)	8 (24)	11 (69)	21 (64)
Thavendiranathan et al. (2021) [33]	86	MACE*: cardiovascular death, cardiogenic shock, cardiac arrest, and complete heart block	NA	NA	NA	NA	67 (78)	67 (78)	NA	27 (31.4)
Zhang et al. (2020) [18]	103	MACE*: cardiovascular death, cardiac arrest, cardiogenic shock, and complete heart block (CHB) requiring a pacemaker	49	41	19	22	NA	NA	NA	NA
Greulich et al. (2020) [19]	183	All-cause death, cardiac death, and sudden cardiac death (SCD)**	101	111	55	46	NA	NA	NA	NA
Gräni et al. (2019).1 [20]	670	MACE**: all-cause death, heart failure (HF) decompensation requiring hospital admission, heart transplantation (HT), documented sustained ventricular arrhythmia (> 30 s), recurrent AM	294	98	66	30	NA	NA	NA	NA
Gräni et al. (2019).2 [21]	179	MACE**: all cause death, HF decompensation requiring hospital admission, HT, documented sustained ventricular arrhythmia (> 30 s), recurrent AM	79	22	14	8	NA	NA	50 (28)	22
Lee et al. (2017) [22]	37	MACE**: cardiac death, HT, implanted cardioverter defibrillator (ICD) or pacemaker, rehospitalization following a cardiac event, or embolic stroke	23	11	10	1	NA	NA	NA	NA
Spieker et al. (2017) [23]	46	MACE**: all-cause death, cardiac death, HT and ventricular assist device implantation	37	11	10	1	NA	46 (100)	NA	11
Aquaro et al. (2017) [24]	374	MACE**: all-cause death; HF decompensation requiring hospital admission, HT, documented sustained ventricular arrhythmia (>30), recurrent AM	348	29	29	0	NA	NA	NA	NA
Chopra et al. (2016) [25]	88	MACE**: all-cause mortality, cardiac mortality, recurrence of AM, HF, and sustained ventricular tachycardia	14	21	3	18	NA	NA	NA	NA
Schumm et al. (2014) [26]	405	MACE**: cardiac death, sudden cardiac death, aborted SCD	114	13	11	2	NA	NA	NA	NA
Grün et al (2012) [27]	203	All-cause death, cardiac death, SCD**	108	39	37	2	NA	NA	NA	NA

*MACE is defined as a composite of cardiovascular death (including sudden death), sustained ventricular tachycardia, ventricular fibrillation, complete atrioventricular heart block requiring a pacemaker, and cardiogenic shock

** The composite endpoint is defined as MACE and/or one of the following endpoints: all-cause death, HF decompensation requiring hospitalization, HT, and recurrent AM

**Table 2b:** Summary of studies with available prognostic data in relation to the presence of LGE on CMR and parametric mapping parameters

First author, study year	No of patients	Endpoints	LGE (+), *n*	Total *n* of events	Total number of events, LGE (+)	Total number of events, LGE (-)	T1 elevated, *n* (%)	T2 elevated, *n* (%)	ECV elevated, *n* (%)	Total *n* of events (%)
Vos et al. (2022) [28]	162	MACE**: all-cause mortality, HT, HF hospitalizations, and life-threatening arrhythmias	145	29	24	5	NA	NA	NA	NA
Ghionzoli et al. (2022) [29]	61	Composite endpoint of all-cause mortality and hospitalization for HF**	57	7	5	2	NA	NA	NA	NA
Wang et al. (2025) [30]	235	MACE**: cardiac death, HF hospitalization, HT, sustained ventricular arrhythmia, and recurrent AM	162	45	36	9	*	*	*	45(19)
Aquaro et al. (2026) [31]	210	Cardiac death, resuscitated cardiac arrest, appropriate ICD shock, HF hospitalization and myocarditis recurrence**	162	31	31	0	NA	NA	NA	NA

*The numerical value of the parameter is given

**The composite endpoint defined as MACE and/or one of the following endpoints: all-cause death, HF decompensation requiring hospitalization, HT, and recurrent AM

### Statistical analysis: all studies

#### Pooled estimates

**Fig. 2:**
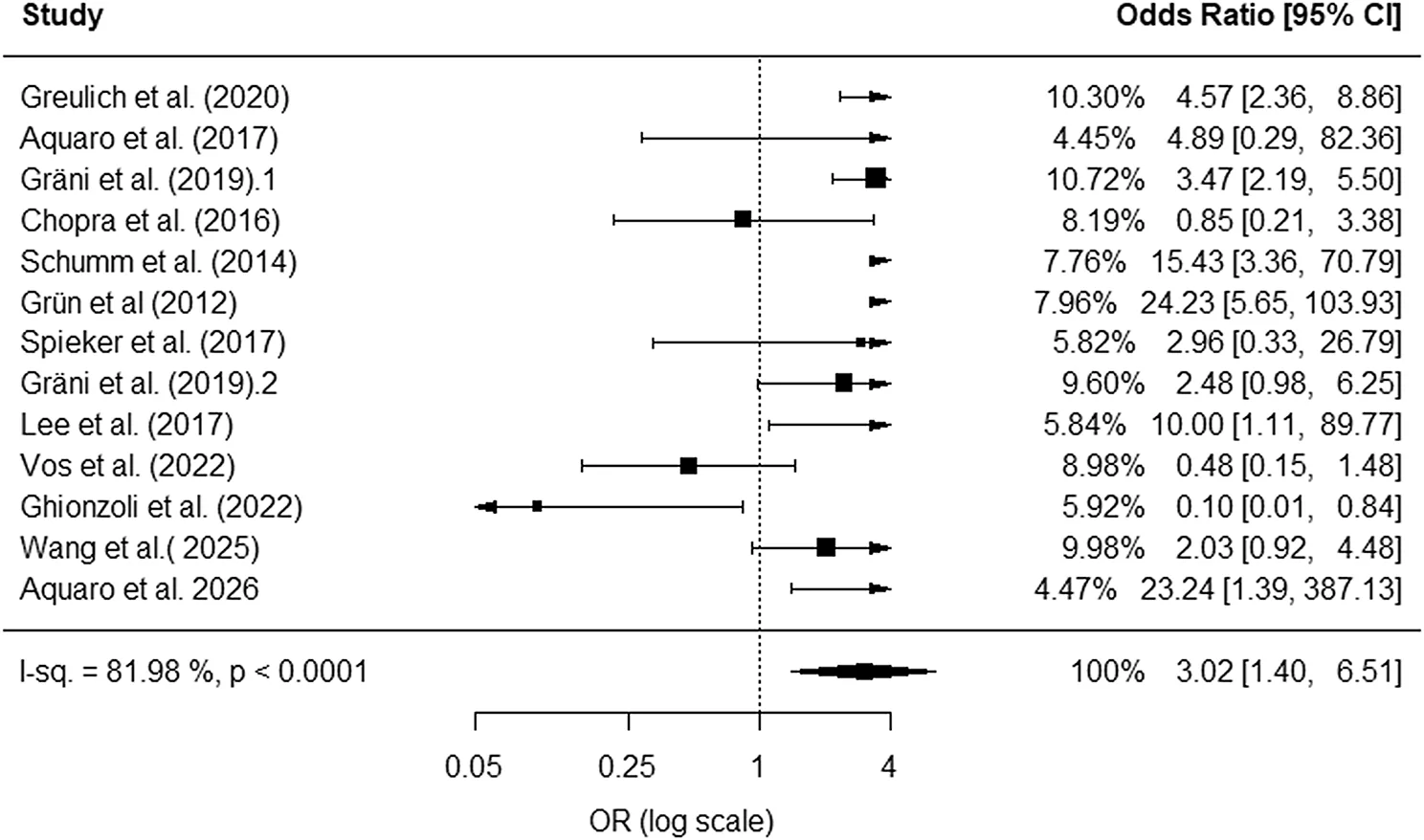
Forest plot spanning all 13 studies selected for meta-analysis (percentages preceding ORs show studies’ weights)

Pooled OR estimates were derived from 2 × 2 tables representing event counts. Adjusted ORs were not used for pooling in this analysis because of: (1) varying sets of covariates and (2) different methodological approaches employed by different studies for estimation of ORs. The presence of LGE was significantly associated with higher odds of the composite endpoint: pooled (OR_LGE(+) vs. LGE(−)_[95% CI] = 3.02 [1.40, 6.51], *p* < 0.001). The corresponding forest plot is presented in Fig. 2.

**Fig. 3:**
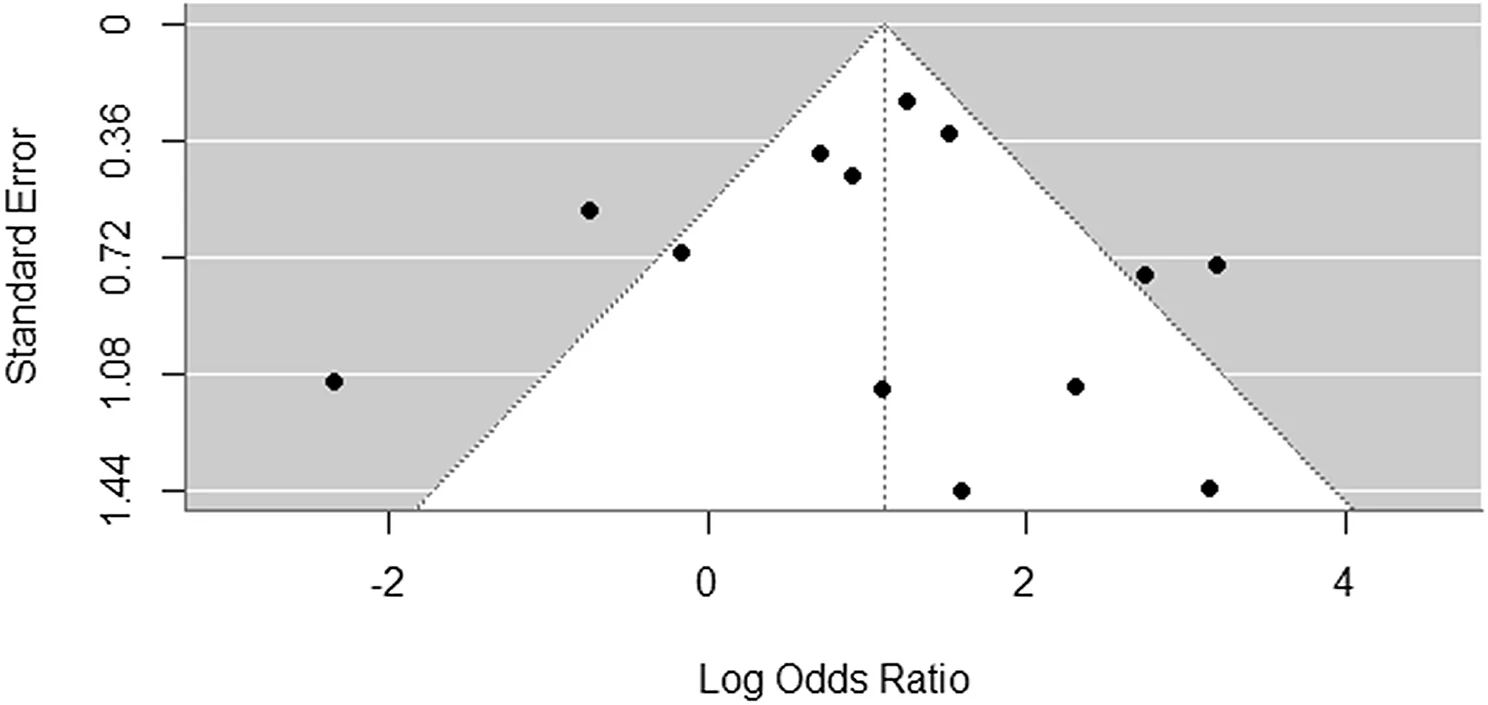
Funnel plot for assessment of heterogeneity in all 13 studies selected for meta-analysis

Heterogeneity was high (I^2^ = 81.98%, *p* < 0.0001) and its presence was also confirmed by the funnel plot (see Fig. 3): some points fell outside the triangle of pseudo 95% confidence limits [34].

#### Publication bias

To assess publication bias, two tests based on the asymmetry of the funnel plot were conducted. The first test had a standard error as an independent variable, whereas the second one had a sample size as an independent variable. Both tests employed a weighted regression with a multiplicative dispersion term [17]. The corresponding p-values are reported in Table 3. No clear evidence of publication bias was detected; however, power to detect small-study effects is limited and results should be interpreted cautiously.

**Table 3:** Linear regression tests for publication bias in all 13 studies selected for meta-analysis

Independent variable	*P* value for Regression Test for Funnel Plot Asymmetry
Standard error	0.9510
Sample size	0.4834

#### Cumulative meta-analysis

**Fig. 4:**
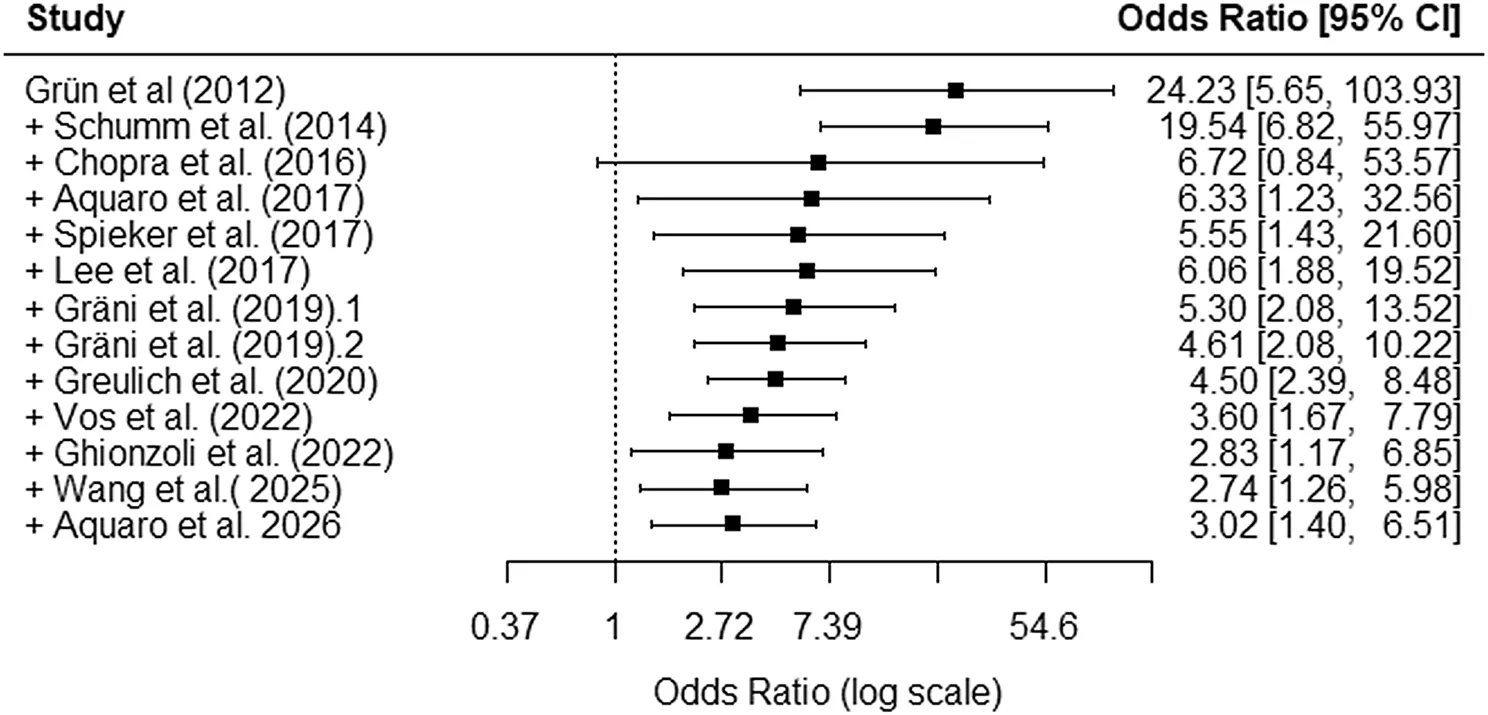
Cumulative forest plot spanning all 13 studies selected for meta-analysis

To investigate the temporal evolution of effect estimates observed in different studies, a cumulative meta-analysis was performed. Its results are summarized in the cumulative funnel plot given in Fig. 4.

As the plot suggested, there was a quite stable downgrade towards the final estimate pooled from all studies. Therefore, we additionally investigated the Mixed Effects model with a study year (in the form of offset starting from 2012) as a moderator. The study year did not appear to be a significant moderator (*p* = 0.0697).

#### Sensitivity analysis

We have also performed a leave-one-out analysis. The results are discussed in the next subsection.

### Additional statistical analysis: 8 studies’ subset

#### Analysis for heterogeneity mitigation

As reported previously, in the set of all 13 selected studies, the large I^2^ = 81.98% (*p* < 0.0001) indicated presence of high heterogeneity (Fig. 2). Leave-one-out analysis did not enable us to locate influential studies causing observed heterogeneity (Table S2). Therefore, we decided to resort to analysis of subgroups and excluded four studies (Greulich et al. [19], Grün et al. [27], Ghionzoli et al. [29], and Schumm et al. [26]) focusing on broader endpoints (MACE and all-cause death). Though in the remaining 9 studies’ cohort the total heterogeneity index decreased significantly, its level still was high (I^2^ = 54.90%, *p* = 0.0302; Figure S1). However, this time subsequent leave-one-out analysis revealed that there remained only one study responsible for a high heterogeneity (Table S3) and removal of this study (Vos et al. [28]) would result in a homogeneous studies’ population with I^2^ = 3.52%, *p* = 0.3358. The remaining 5 studies’ population was extremely heterogeneous (I^2^ = 91.99%, *p* < 0.0001). Though point pooled effect estimate in this subgroup indicated a statistically significant association between LGE and composite event, association was not significant: OR_LGE(+) vs. LGE(−)_[95% CI] = 2.56 [0.36, 18.21], *p* = 0.3469 (for a complete view, see forest plot in Figure S2). Subdividing this already small subgroup further was not feasible because of sample size deflation. Trying to fit regression model with subgroup indicator also did not produce desired result: residual heterogeneity remained high and significant (I^2^ = 82.54%, *p* < 0.0001) and inclusion of additional study year moderator did not resolve the problem (I^2^ = 76.49%, *p* = 0.0004). Therefore, we ended up with a homogeneous subgroup of 8 studies having MACE as a primary endpoint. Subsequent analyses presented below were conducted for that group.

#### Subsequent analyses

For the located 8 studies homogeneous subgroup, we replicated the analyses conducted for the whole 13 studies group:

1. Association of presence of LGE was estimated by computing pooled unadjusted OR_LGE(+) vs. LGE(-)_[95% CI] = 2.88 [2.01, 4.13], *p* < 0.0001 (the corresponding forest plot is presented in Figure S3).
2. Absence of heterogeneity, indicated by small by I^2^ = 3.52% (*p* = 0.3358), was additionally investigated and confirmed by the funnel plot (see Figure S4 showing that there were no points falling outside the triangle of pseudo 95% confidence limits).
3. Two funnel plot asymmetry-based tests employing a weighted regression with a multiplicative dispersion term were performed (Table S4) with an aim to assess publication bias: neither of them suggested its presence.
4. Temporal evolution of effect estimates was assessed by means of a cumulative meta-analysis (Figure S5) and Mixed Effects model with a study year as a moderator. The latter confirmed that, as in the whole cohort case, the study year did not act as a significant moderator (*p* = 0.8284).
5. To determine whether removing any of the studies used affects the results of this subgroup meta-analysis, leave-one-out analysis was done (Table S5). It revealed that there were no sensitivity issues.

Moreover, we computed prediction interval (PI) for the pooled OR to see if there were any evidence to observe non-significant cumulative effect soon. The obtained result was in line with the ones stated above: OR_LGE(+) vs. LGE(−)_[95% PI] = 2.88 [1.89, 4.38].

### Prognostic value of CMR parametric mapping

The characteristics of the studies analysing parametric mapping parameters are summarised in Table 2a, b.

### Native T1 mapping

There are limited data on T1 mapping prognostic value in regards of MACE in AM. An increased T1 time is thought to be associated with a worse prognosis, but the results are controversial. In our search, only three studies reported the prognostic value of native T1 mapping [30, 32, 33], and the results are conflicting. Two studies included patients with ICI - myocarditis. The sample sizes of the above-mentioned studies were 33 and 86 participants [32, 33] and abnormal T1 value has been found in 36% and 78% of patients, respectively. Cadour et al. [32] concluded that T1 mapping doesn’t predict MACE at 1-year. In Thavendiranathan et al. [33] study, native T1 values had excellent discriminatory capacity for subsequent MACE, with an area under the receiver operator curve (ROC) equal to 0.91. Wang et al. [30] latest study sample was relatively large, including 235 patients with AM whose diagnosis was confirmed with cardiac MRI or biopsy. It was concluded that global native T1 values were significantly higher in the group with MACEs than in the group without MACEs and T1 was independently associated with MACEs.

### T2 mapping

The impact of T2 time on the prognosis of AM is complex and controversial. Four studies analysed the prognostic value of T2 mapping in AM [23, 30, 32, 33]. Two of them included patients with ICI – myocarditis. They showed that patients with AM had significantly higher T2 values than control groups. Although T2 values were also associated with MACE, this association lost significance after adjusting for cardiovascular risk factors and LVEF. Cadour et al. [32] and Thavendiranathan et al. [33] did not establish T2 prognostic value on MACE. Spieker et al. [23] study showed elevated global and regional T2 values that predicted MACE and rehospitalization in patients with suspected AM. Wang et al. [30] study showed T2 values were significantly higher in the group with MACEs compared with the group without MACEs. In univariable Cox regression analyses, T2 was significantly associated with MACEs. However, in the multivariable Cox regression analysis T2 did not show independent prognostic value.

### Extracellular volume (ECV)

Only three included studies evaluated ECV prognostic value in patients with AM [21, 30, 32]. One of them included patients with ICI – myocarditis. Gräni et al. in the study with 179 patients showed that mean ECV (per 10%) was significantly associated with MACE (HR 2.09, 95% CI 1.07–4.08, *p* = 0.031). The presence of ECV ≥ 35% demonstrated a significant univariable association with MACE. However, Cadour et al. noted that global ECV was significantly higher in the ICI - myocarditis group compared with the pre-ICI group but was not a predictor of MACE [32]. Wang et al. [30] study showed ECV was significantly higher in the group with MACEs. In univariable Cox regression analyses, ECV was associated with increased risk of MACEs, and including ECV in addition to native T1 and conventional parameters, had the best prognosis among all models.

## Discussion

This up-to-date systematic review and meta-analysis, incorporating all available evidence published up to January 20, 2026, demonstrates that: (a) the presence of late gadolinium enhancement (LGE) is associated with an adverse prognosis in patients with acute myocarditis (AM) and may contribute to risk stratification; however, given the heterogeneity of the available studies, this finding requires further validation in large prospective cohorts; and (b) the prognostic value of cardiovascular magnetic resonance (CMR)-derived parametric mapping in patients with AM remains uncertain because of the limited and inconsistent available evidence.

### Late gadolinium enhancement

LGE in CMR detects only focal, but not diffuse fibrosis. LGE is helpful not only for the diagnosis of AM but also the presence of LGE is associated with subsequent adverse outcomes. Almost all studies included in our meta-analysis concluded that LGE presence is a risk marker in patients with suspected AM. Analysed studies have shown that patients with suspected AM may be risk-stratified not only by the presence of LGE but also by LGE location, extent, and distribution.

Our meta-analysis did not aim to analyse the prognostic value of LGE extent and localization. In the study by Chopra et al. [25], LGE distribution predominated in the inferolateral and anterolateral (AL) basal segments, followed by the midsegments of the heart. According to Aquaro et al. [24], the AL group had a greater extent of LGE and a higher LV end-diastolic volume index than other groups. LGE in the mid-wall layer of the AS myocardial segment is associated with a worse prognosis than other presentation patterns. Greulich et al. [19] findings were concordant. Also, Aquaro et al. [31] concluded that midwall-septal/ring-like LGE pattern was confirmed to be an important prognostic marker in AM. In patients with biopsy-proven viral myocarditis, mid-wall LGE in septal segments and the extent of LGE were highly associated with death. In the individual study by Grün et al. [27], LGE remained an independent predictor of all-cause mortality and cardiac mortality in viral myocarditis after multivariable adjustment. A threshold of LGE mass > 17 g provided a high discrimination for MACE occurrence, summarized by Chopra et al. [25]. Ghionzoli et al. [29] study with small cohort of patients having myocarditis with preserved left ventricular ejection fraction concluded that both the presence and extent of LGE were predictive of worse outcomes (*p* = 0.022). This association was further confirmed when considering the percentage of LGE mass in the left ventricle relative to total LV mass.

Two studies investigated quantification methods of LGE extent. Gräni et al.1 [20] analysed different semi-automated quantification methods of LGE extent and found LGE (%) quantification by 2-, 3-SD, or FWHM (full width at half maximum) demonstrated robust prognostic association with MACE. Also, visual scoring showed a strong association with outcome. Visual assessment of LGE may aid clinical risk stratification n in a diffuse or patchy disease such as myocarditis [21]. According to Wang et al. [30] the group with MACEs had greater LGE burden with 13.2% LGE of the LV mass versus 3.8% LGE in the group without MACEs. Although the latter studies used quantitative LGE assessment methods, as compared with the rest using visual LGE assessment, we included them in the overall analysis. We acknowledge this methodological heterogeneity as a limitation.

Only one study that included 103 ICI - myocarditis patients conducted by Zhang et al. [18] reported that the presence of LGE was not associated with subsequent MACE. It is important to mention that LGE was present in < 50% of patients in this specific group of patients. We excluded this study of ICI myocarditis from meta-analysis because it represents a distinct clinical condition.

The study performed by Schumm et al. [26] had a different purpose compared to the other studies. The aim was not to identify predictors of adverse events in patients with suspected myocarditis but to establish risk stratification in clinical routine. Results demonstrated that adverse cardiac events are extremely rare in patients with normal CMR, independent of the symptomatic status [26].

Substantial heterogeneity across the initial 13 studies (I² = 81.98%), likely due to differences in patient populations, endpoint definitions, and follow-up duration, represented a major limitation. Sensitivity analyses identified a homogeneous subgroup of eight studies with MACE as the primary endpoint (I² = 3.52%), in which the association between LGE and adverse outcomes remained statistically significant. Nevertheless, these findings should be interpreted cautiously and warrant confirmation in future studies using standardized endpoint definitions and study protocols.

### Native T1 mapping

T1 mapping could permit the detection and quantification of microscopic fibrosis, even in the absence of positive LGE [24]. Cadour et al. [32] compared ICI-myocarditis with the pre-ICI group, and patients with ICI-myocarditis showed higher global native T1, ECV, and T2 *z* scores, and LGE was more frequently observed, but T1 mapping didn’t predict MACE at 1-year. In Thavendiranathan et al. [33] study, patients who developed MACE had higher T1 and T2 z-scores. Native T1 values had excellent discriminatory values for subsequent MACE, with an area under the ROC of 0.91. Gräni et al. [21] study reported that mean native T1 and abnormal native T1 were not associated with outcome, but the proportion of patient elevated T1 was missing. Wang et al. concluded that Cox regression models combining native T1 mapping and ECV with conventional parameters showed improved performance in predicting MACEs compared with the baseline model [30].

The prognostic value of native T1 mapping in predicting MACE in AM is still uncertain due to limited study data, heterogeneous AM populations with different AM clinical entities, and conflicting results. The available data were insufficient to permit a quantitative meta-analysis. Two of three studies included in our analysis had small sample sizes, and the reported prevalence of abnormal T1 values differed significantly between the studies. Contradictory prognostic conclusions were summarized. The divergence in findings may be due to differences in study design, patient populations, or the specific T1 technique used. It is important to emphasize that T1 value strongly depends on the sequence used and is vendor specific. Larger, more comprehensive studies are needed to clarify the role of T1 mapping in risk stratification and prognosis in patients with AM.

### T2 mapping

T2 time is a marker of myocardial inflammation and is significantly elevated at initial presentation, reflecting the acute stage of the disease and declined during the healing process of myocarditis [23]. We found conflicting evidence on T2 mapping prognostic value. As mentioned above in Thavendiranathan et al. [33] study, patients who developed MACE had higher T2 z-scores and T2 values. Although T2 values were also associated with MACE, this lost significance after adjusting for cardiovascular risk factors and LVEF. Cadour et al. [32] also did not establish T2 prognostic value for MACE. Supporting evidence was found only in one study. Only Spieker et al. [23] study, including 45 patients, showed elevated global and regional T2 values that predicted MACE and rehospitalization in patients with suspected AM, indicating some potential of T2 mapping for risk stratification. As noted by Wang et al. [30], the dynamic pathophysiology of myocardial edema in AM may explain why the T2 value was an independent prognostic factor in the univariable Cox analysis but not in the multivariable analysis, and further studies are still needed.

The overall evidence on the prognostic value of T2 mapping in AM is mixed. While Spieker et al. [23] suggest a possible role for T2 mapping in predicting MACE, the larger studies by Thavendiranathan et al. [33] and Cadour et al. [32] do not support T2 as an independent predictor of MACE after adjusting for other risk factors. The findings should not be generalized because of heterogeneity in patient populations across studies. More extensive studies are required to establish the role of T2 mapping in predicting MACE in patients with AM. Current evidence is not robust enough to conclusively determine its prognostic utility.

### Extracellular volume (ECV)

The ECV technique introduces a potentially important new method to examine the myocardium because it is sensitive to the distribution of the LV myocardium into its cellular (dominated by myocyte mass) and extracellular interstitial (extracellular matrix (ECM)) compartments. Therefore, ECV characterizes myocardial fibrosis [21]. Gräni et al. [21] showed that mean ECV (per 10%) was significantly associated with MACE with a hazard ratio (HR) of 2.09. This suggests that higher ECV values are indicative of a worse prognosis. Additionally, an ECV value of 35% or higher was significantly associated with MACE in an univariable analysis, further supporting its potential role as a prognostic marker. However, Cadour et al. [32] noted that global ECV was significantly higher in the ICI-M group compared with the pre-ICI group but was not a predictor of MACE, indicating that its prognostic value might not be consistent across different patient populations or might be influenced by other factors.

Given the conflicting results, more studies are needed to clarify the role of ECV in predicting MACE, particularly across different types of myocarditis and patient populations. The potential for ECV to serve as a reliable prognostic marker is promising but not yet definitively established.

### Comparison with other metanalyses and systematic literature reviews

In the PubMed and Scopus databases, we found a few systematic literature reviews and meta-analyses performed and spanning years from 2019 until 2023. Previous reviews support the results of our analysis. S. Blissett et al. [35] meta-analysis (studies from 2005 until 2017), which included 2823 myocarditis patients and 803 controls, concluded that the presence of baseline LV dysfunction and LGE predict patients at higher risk of adverse events. After controlling for confounding, only LGE and baseline LVEF remained independent predictors of adverse clinical events in adjusted analyses. F. Yang et al. [36] meta-analysis (studies from 2012 until 2019) of eight studies showed that positive LGE was strongly associated with an increased risk of combined outcome irrespective of LVEF. After 3 years of follow-up, the worst outcomes occurred mainly in patients with positive LGE. G. Georgiopoulos et al. [37], including studies from 2012 until 2018, summarized that LGE presence and anteroseptal location at baseline CMR are important independent prognostic markers that herald an increased risk of adverse cardiac outcomes in patients with AM. W. Song et al. [38] meta-analysis included four cohort studies of patients with ICI - myocarditis and concluded that patients with MACE had a higher incidence of LGE than patients without MACE.

We performed a meta-analysis of all inclusion/exclusion criteria meeting studies up to January 20th 2026 with relatively long follow-up. Additionally, we analyzed all available studies on the prognostic value of parametric mapping. While there is a substantial amount of data on LGE, the prognostic value of parametric mapping techniques like T1, T2, and ECV is less well-established. No meta-analyses are present at this point on the prognostic value of parametric mapping in AM, so performing further research would help to fill this gap and provide a clearer understanding of their prognostic utility in AM.

### Other CMR techniques carrying potential for risk stratification

Emerging evidence underscores the increasing clinical importance of left ventricular strain imaging for the early detection of AM, particularly in patients with preserved left ventricular ejection fraction. Feature-tracking CMR has demonstrated high sensitivity for identifying subtle myocardial dysfunction in the acute phase, providing incremental diagnostic value beyond conventional functional parameters [39]. Similarly, speckle-tracking echocardiography enables the detection of early contractile abnormalities that may precede overt reductions in systolic function and has shown promise as an accessible bedside tool for myocardial assessment [40]. Beyond functional evaluation, speckle-tracking echocardiography also carries translational relevance for tissue characterization, as strain-derived parameters correlate strongly with CMR-based measures of myocardial fibrosis burden [41]. Collectively, these findings support the complementary role of strain imaging modalities in the acute evaluation of myocarditis and highlight their potential utility for both early diagnosis and risk stratification.

### Limitations

Our systematic literature review and meta-analysis have several limitations. First, we included studies with small sample sizes, which may limit the generalizability of our findings. Additionally, the studies varied in their definition of endpoints and the duration of follow-up, introducing potential inconsistencies in outcome assessment. We made an attempt to explore the observed heterogeneity in prognostic association of LGE with statistical means which ended up in reduction of studies employed in the additional meta-analysis. Due to observed heterogeneity, our findings should be interpreted with some caution and future studies with more robust design are needed to provide more consistent conclusions. Because the pooled analysis was based exclusively on unadjusted event counts, the observed association between LGE and adverse outcomes should not be interpreted as evidence of an independent prognostic effect.

CMR imaging was performed at different times in respect to symptom onset, and variations in magnet strength further complicate comparisons across studies. Furthermore, only a small percentage of cases were biopsy-proven AM, which may affect the accuracy of diagnosis. We also did not analyze data on the clinical characteristics of the included patients, nor did we assess the impact of clinical utility and accuracy of T1 and T2 mapping across different vendor-specific sequences. Additionally, we acknowledge that the use of different LGE quantification methods across studies represents a potential source of heterogeneity and constitutes a limitation of the present analysis.

An important limitation of this meta-analysis is the heterogeneity of clinical endpoints across the included studies as well as different methodological approaches employed by different studies for estimation of ORs. Differences in endpoint definitions—such as MACE versus composite outcomes including hospitalization or recurrent myocarditis—may have influenced the pooled prognostic estimates and their clinical interpretation. This variability should be considered when extrapolating our findings to clinical practise.

## Conclusion

Based on the findings, the presence of LGE appears to be associated with an increased risk of adverse clinical outcomes in patients with AM. However, these findings should be interpreted with caution because of substantial heterogeneity among studies and the predominantly unadjusted effect estimates. There is very limited and discrepant data on the prognostic value of CMR parametric mapping in AM. Large prospective studies are needed to assess the additive prognostic value of parametric mapping besides LGE in patients with AM.

## Supplementary Information

Below is the link to the electronic supplementary material.

Supplementary Material 1

## Data Availability

All data analysed during this study are included in this published article.

## Abbreviations

AM: Acute myocarditis
AL: Anterolateral
CI: Confidence interval
CMR: Cardiovascular magnetic resonance
ECV: Extracellular volume
EMB: Endomyocardial biopsy
FE: Fixed effects
FWHM: Full width at half maximum
ICI: Myocarditis–Immune Checkpoint Inhibitor–Associated Myocarditis
LGE: Late gadolinium enhancement
MACE: Major adverse cardiovascular events
OR: Odds ratio
PI: Prediction interval
RE: Random effects
ROC: Receiver operator curve
SCD: Sudden cardiac death
