# Supplementary material for: Prognostic value of cardiovascular magnetic resonance in acute myocarditis: a systematic literature review and meta-analysis of late gadolinium enhancement and narrative literature review of parametric mapping
Source: BMC Med Imaging. 2026 Jul 31;26:568. doi: 10.1186/s12880-026-02623-6 (PMC13729655; doi:10.1186/s12880-026-02623-6)
Supplement: Supplementary file 1 [file 12880_2026_2623_MOESM1_ESM.docx]

Supplementary material

**Table S1. Quality assessment using Newcastle Ottawa scale.**

| First author,  study year | **Representativeness**  **of the exposed**  **cohort**  **0-1** | **Selection of the non-exposed cohort**  **0-1** | **Ascertainment of exposure**  **0-1** | **Demonstration that outcome of interest was not present at the start of the study**  **0-1** | **Comparability of cohorts based on the design or analysis**  **0-2** | **Assessment of outcome**  **0-1** | **Was follow-up long enough for outcomes to occur**  **0-1** | **Adequacy of**  **follow-up**  **0-1** | | **Total punctuation**  **0-9** |
| --- | --- | --- | --- | --- | --- | --- | --- | --- | --- | --- |
| Greulich et al. (2020) [19] | 1 | 1 | 1 | 1 | 0 | 1 | 1 | 1 | | 7 |
| Gräni et al. (2019).1 [20] | 1 | 1 | 1 | 1 | 1 | 1 | 1 | 1 | | 8 |
| Gräni et al. (2019).2 [21] | 1 | 1 | 1 | 1 | 1 | 1 | 1 | 1 | | 8 |
| Lee et al. (2017) [22] | 1 | 1 | 1 | 1 | 1 | 1 | 0 | 0 | | 6 |
| Spieker et al. (2017) [23] | 1 | 1 | 1 | 1 | 1 | 1 | 1 | 1 | | 8 |
| Aquaro et al. (2017) [24] | 1 | 1 | 1 | 1 | 1 | 1 | 1 | 1 | | 8 |
| Chopra et al. (2016) [25] | 1 | 1 | 1 | 1 | 1 | 1 | 0 | 0 | | 6 |
| Schumm et al. (2014) [26] | 1 | 1 | 1 | 0 | 1 | 1 | 1 | 1 | | 7 |
| Grün et al. (2012) [27] | 1 | 1 | 1 | 1 | 1 | 1 | 1 | 1 | | 8 |
| Vos et al. (2022)[28] | 1 | 1 | 1 | 1 | 2 | 1 | 1 | 1 | | 9 |
| Ghionzoli et al. (2022)[29] | 1 | 1 | 1 | 1 | 1 | 1 | 1 | 1 | | 8 |
| Wang et al. (2025)[30] | 1 | 1 | 1 | 1 | 1 | 1 | 0 | 1 | | 7 |
| Aquaro et al. (2026)[31] | 1 | 1 | 1 | 1 | 2 | 1 | 1 | 1 | | 9 |

Each included study was assessed using the Newcastle–Ottawa Scale (NOS) across eight domains: representativeness of the exposed cohort, selection of the non-exposed cohort, ascertainment of exposure, demonstration that outcome was not present at baseline, comparability of cohorts, assessment of outcome, follow-up duration, and adequacy of follow-up. Each domain is scored 0–1 (comparability 0–2), with higher scores indicating lower risk of bias.

**Table S2. Leave one out analysis for all 13 initially selected studies.**

| **Study removed** | **OR [95% CI]** | **OR p-value** | **I^2^ (p-value)** |
| --- | --- | --- | --- |
| -Greulich et al. (2020) | 2.90 [1.22, 6.86] | 0.0156 | 81.87 (<.0001) |
| -Aquaro et al. (2017) | 2.95 [1.31, 6.65] | 0.0089 | 84.16 (<.0001) |
| -Gräni et al. (2019).1 | 2.99 [1.25, 7.14] | 0.0139 | 79.94 (<.0001) |
| -Chopra et al. (2016) | 3.38 [1.51, 7.59] | 0.0032 | 82.66 (<.0001) |
| -Schumm et al. (2014) | 2.62 [1.21, 5.70] | 0.0146 | 81.20 (0.0001) |
| -Grün et al (2012) | 2.50 [1.22, 5.12] | 0.0121 | 77.54 (0.0004) |
| -Spieker et al. (2017) | 3.03 [1.32, 6.94] | 0.0087 | 84.48 (<.0001) |
| -Gräni et al. (2019).2 | 3.10 [1.31, 7.34] | 0.0103 | 83.77 (<.0001) |
| -Lee et al. (2017) | 2.80 [1.25, 6.28] | 0.0123 | 83.55 (<.0001) |
| -Vos et al. (2022) | 3.60 [1.73, 7.48] | 0.0006 | 77.55 (0.0012) |
| -Ghionzoli et al. (2022) | 3.62 [1.84, 7.12] | 0.0002 | 75.78 (0.0008) |
| -Wang et al. (2025) | 3.16 [1.34, 7.49] | 0.0088 | 82.98 (<.0001) |
| -Aquaro et al. (2026) | 2.74 [1.26, 5.98] | 0.0112 | 82.69 (<.0001) |

**Table S3. Leave one out analysis for 9 studies having MACE as a primary endpoint.**

| **Study removed** | **OR [95% CI]** | **OR p-value** | **I^2^ (p-value)** |
| --- | --- | --- | --- |
| -Aquaro et al. (2017) | 2.19 [1.16, 4.15] | 0.0161 | 59.59 (0.0189) |
| -Gräni et al. (2019).1 | 2.04 [0.98, 4.22] | 0.0550 | 48.85 (0.0676) |
| -Chopra et al. (2016) | 2.55 [1.33, 4.91] | 0.0049 | 56.33 (0.0430) |
| -Spieker et al. (2017) | 2.24 [1.15, 4.34] | 0.0176 | 61.38 (0.0176) |
| -Gräni et al. (2019).2 | 2.28 [1.07, 4.85] | 0.0327 | 62.59 (0.0174) |
| -Lee et al. (2017) | 2.06 [1.11, 3.80] | 0.0216 | 55.17 (0.0308) |
| -Vos et al. (2022) | 2.88 [2.01, 4.13] | <0.0001 | 3.52 (0.3358) |
| -Wang et al. (2025) | 2.38 [1.10, 5.14] | 0.0279 | 61.43 (0.0197) |
| -Aquaro et al. (2026) | 2.05 [1.13, 3.73] | 0.0184 | 54.09 (0.0422) |

**Table S4. Linear regression tests for publication bias for 8 studies selected for additional analysis.**

| **Independent variable** | **P value for Regression Test for Funnel Plot Asymmetry** |
| --- | --- |
| Standard error | 0.7207 |
| Sample size | 0.3331 |

**Table S5. Leave one out analysis for 8 studies selected for additional analysis.**

| **Study removed** | **OR [95% CI]** | **OR p-value** | **I^2^ (p-value)** |
| --- | --- | --- | --- |
| -Aquaro et al. (2017) | 2.84 [1.95, 4.14] | <0.0001 | 6.31 (0.2506) |
| -Gräni et al. (2019).1 | 2.37 [1.43, 3.91] | 0.0008 | 0.00 (0.3445) |
| -Chopra et al. (2016) | 3.15 [2.22, 4.48] | <0.0001 | 0.00 (0.5813) |
| -Spieker et al. (2017) | 2.86 [1.95, 4.19] | <0.0001 | 6.52 (0.2407) |
| -Gräni et al. (2019).2 | 2.89 [1.80, 4.64] | <0.0001 | 14.37 (0.2510) |
| -Lee et al. (2017) | 2.75 [1.86, 4.05] | <0.0001 | 7.82 (0.3474) |
| -Wang et al. (2025) | 3.16 [2.17, 4.60] | <0.0001 | 0.00 (0.3222) |
| -Aquaro et al. (2026) | 2.75 [1.88, 4.03] | <0.0001 | 7.06 (0.4414) |

**Figure S1.** Forest plot spanning a subset of 9 studies focusing on MACE (percentages preceding ORs show studies’ weights).

**Figure S2.** Forest plot spanning 5 heterogeneity causing studies discarded from subsequent analysis in “Additional Statistical Analysis” subsection (percentages preceding ORs show studies’ weights).

**Figure S3.** Forest plot for estimated pooled ORLGE(+) vs LGE(-) in the cohort of 8 studies selected for additional analysis (percentages preceding ORs show studies’ weights).

**Figure S4.** The funnel plot for assessment of heterogeneity in a homogeneous cohort consisting of 8 studies selected for additional analysis.

**Figure S5**. Cumulative meta-analysis forest plot in a homogeneous cohort consisting of 8 studies selected for additional analysis.
